# Clinical Features to Predict the Use of a sEMG Wearable Device (REMO^®^) for Hand Motor Training of Stroke Patients: A Cross-Sectional Cohort Study

**DOI:** 10.3390/ijerph20065082

**Published:** 2023-03-14

**Authors:** Giorgia Pregnolato, Daniele Rimini, Francesca Baldan, Lorenza Maistrello, Silvia Salvalaggio, Nicolò Celadon, Paolo Ariano, Candido Fabrizio Pirri, Andrea Turolla

**Affiliations:** 1 Laboratory of Healthcare Innovation Technology, IRCCS San Camillo Hospital, Via Alberoni 70, 30126 Venice, Italy; lorenza.maistrello@hsancamillo.it (L.M.); silvia.salvalaggio@hsancamillo.it (S.S.); 2 Medical Physics Department, Salford Care Organisation, Northern Care Alliance, Salford M6 8HD, UK; daniele.rimini@nca.nhs.uk; 3 Division of Cardiovascular Sciences, School of Medical Sciences, Faculty of Biology, Medicine and Health, University Of Manchester, Manchester M13 9PL, UK; 4 FisioSPORT Terraglio s.r.l., 30174 Venezia, Italy; fra.bald@hotmail.it; 5 Padova Neuroscience Center, Università degli Studi di Padova, Via Orus 2/B, 35131 Padova, Italy; 6 Morecognition s.r.l., 10129 Turin, Italy; nicolo.celadon@gmail.com (N.C.); paolo@morecognition.com (P.A.); 7 Artificial Physiology Group, Center for Sustainable Future Technologies, Istituto Italiano di Tecnologia, Via Livorno 60, 10144 Torino, Italy; fabrizio.pirri@iit.it; 8 Department of Applied Science and Technology, Politecnico di Torino, Corso Duca degli Abruzzi 24, 10129 Turin, Italy; 9 Department of Biomedical and Neuromotor Sciences—DIBINEM, Alma Mater Studiorum Università di Bologna, Via Massarenti, 9, 40138 Bologna, Italy; andrea.turolla@unibo.it; 10 Unit of Occupational Medicine, IRCCS Azienda Ospedaliero-Universitaria di Bologna, Via Pelagio Palagi, 9, 40138 Bologna, Italy

**Keywords:** neurological rehabilitation, upper extremity, wearable technology, surface electromyography, myoelectric control, hand gesture

## Abstract

After stroke, upper limb motor impairment is one of the most common consequences that compromises the level of the autonomy of patients. In a neurorehabilitation setting, the implementation of wearable sensors provides new possibilities for enhancing hand motor recovery. In our study, we tested an innovative wearable (REMO^®^) that detected the residual surface-electromyography of forearm muscles to control a rehabilitative PC interface. The aim of this study was to define the clinical features of stroke survivors able to perform ten, five, or no hand movements for rehabilitation training. 117 stroke patients were tested: 65% of patients were able to control ten movements, 19% of patients could control nine to one movement, and 16% could control no movements. Results indicated that mild upper limb motor impairment (Fugl-Meyer Upper Extremity ≥ 18 points) predicted the control of ten movements and no flexor carpi muscle spasticity predicted the control of five movements. Finally, severe impairment of upper limb motor function (Fugl-Meyer Upper Extremity > 10 points) combined with no pain and no restrictions of upper limb joints predicted the control of at least one movement. In conclusion, the residual motor function, pain and joints restriction, and spasticity at the upper limb are the most important clinical features to use for a wearable REMO^®^ for hand rehabilitation training.

## 1. Introduction

Stroke is one of the leading causes of disability worldwide [1,2,3]. The global prevalence of cerebrovascular disorders was around 80 million of people in 2016, and in the United States, prevalence is expected to increase to 20.5% compared to 2012 [4,5,6,7]. Indeed, American projections show that by 2030, an additional 3.4 million adults ≥18 years of age will be diagnosed with a stroke disease [3].

After a cerebrovascular lesion, almost 85% of stroke survivors experience impairment of the motor function [8,9] and nearly 70% lose independence in their daily living activities [10]. Moreover, recent studies found that after six months following a brain lesion, 30% to 66% of stroke survivors still experience impairment of the upper limb [10]. For all these people, the disabling condition seriously affects their quality of life [11,12].

In stroke rehabilitation, the reduction of motor impairments and the promotion of social participation remain challenging goals for both clinicians and researchers [13]. By achieving these rehabilitation goals, researchers highlight the importance of early assessment of the sensory-motor impairments of stroke survivors [13,14,15,16] to provide the most appropriate evidence-based rehabilitation programs [17,18]. For upper limb recovery especially, the early and focus-specific assessment of motor and sensory functions is fundamental to refer to different rehabilitation approaches [19]. Moreover, recent studies confirmed that the patient clinical features assessed at the baseline predicted the final outcomes of the motor treatment [20,21]. Nevertheless, more detailed stratification based on cut-off scores of the upper limb clinical scale are still lacking.

In the neurorehabilitation approach, technology-based rehabilitation (TBR) merged as a clinical modality that provides new opportunities to enhance motor recovery after stroke [22,23]. Indeed, the last literature overviews confirm that the use of technological solutions, such as virtual reality or robotic devices, have shown encouraging results regarding motor recovery in stroke survivors. However, there is a lack of evidence to support which approach is better [22,23,24,25].

The use of TBR for upper limb motor recovery in stroke rehabilitation is still under investigation. In their work, Everard et al. affirmed that virtual reality is more effective both in sub-acute and chronic phases of stroke, robotic-assistive therapy is more effective in patients with severe-moderate motor impairments, and telerehabilitation effectiveness is globally equivalent to conventional therapy when delivered to patients with mild to moderate motor impairments [23]. These results suggest the need to stratify patients according to their clinical features in order to plan for the most appropriate TBR treatment [23,26,27].

Among the options offered by TBR, devices providing biofeedback from surface electromyography (sEMG) represent a reliable solution to enhance motor recovery in stroke rehabilitation [28,29]. Indeed, sEMG-based solutions trigger an interaction with the external environment (e.g., a computer interface) and provide the patients with real-time auditory or visual information on the timing and amplitude of their muscle activation [29,30].

In their work, Munoz-Novoa et al. investigated different sEMG applications in stroke rehabilitation and defined the effect of using these technologies for upper limb motor recovery [31]. Results reported statistically significant improvements in Fugl–Meyer Upper Extremity scores after sEMG training, especially in individuals with chronic and severe stroke motor impairment [31]. Nevertheless, more trials using sEMG biofeedback in upper limb motor recovery are needed to determine its effectiveness compared with other interventions [31].

In the last years, the use of electromyography biofeedback applied to wearable devices is growing in the field of stroke rehabilitation for real-time monitoring of muscle activations and as a controller of human-machine interfaces [32,33]. Recently, Meeker et al. used sEMG to drive a hand orthosis for functional grasp in stroke survivors. They integrated a commercial sEMG wearable with an exotendon device for closing and opening of the hand, then asked a sample of stroke survivors to control the device [34]. Intention to move was detected through application of sEMG pattern recognition at the forearm muscles. In this way, the sensory-motor loop was closed, allowing the patient to control the device. Nevertheless, the clinical features of patients able to exploit this approach were not investigated and similar information is still missed in the literature [34].

In this study, we developed a sEMG-controlled wearable (REMO^®^, Morecognition s.r.l., Torino, Italy) able to detect muscle activation of the forearm muscles. Due to its capability of transducing muscle activation in hand gestures, the device may be employed in a clinical setting to detect specific hand and fingers movements to control a machine interface in an augmented rehabilitative environment. Specifically, in a few seconds, the device can classify sEMG of hand gestures, regardless of the kinematics, using a specific algorithm developed for muscle patterns recognition [35]. In a previous work, we tested REMO^®^ to define its safety and feasibility in a clinical setting on stroke patients and its efficacy in extracting quantitative parameters of subjects’ hand performance [35]. Furthermore, in our last work, we tested REMO^®^ device in a longitudinal pilot study with the aim to define the clinical effect in specific hand motor training in stroke survivors [36]. Preliminary data defined that there was a significant improvement in upper limb motor performance correlated with an improvement of the number of controlled movements required in the training [36].

To date, the clinical features of stroke survivors who are able to use REMO^®^ is still lacking. For this reason, the aim of this cross-sectional cohort study is to define the clinical features of stroke patients and the cut-off scores to predict the ability to use REMO^®^ in three different conditions of ability (i.e., ten, five, or zero hand movements performed). In this way, we stratified patients based on clinical features to tailor specific hand motor training provided by the REMO^®^ device.

## 2. Materials and Methods

### 2.1. Participants

From July 2017 to February 2019, consecutive survivors of a first stroke episode hospitalized at the Cerebrovascular Unit of the San Camillo IRCCS hospital (Venice, Italy) were clinically assessed and instrumentally tested using the sEMG-control device REMO^®^. Each patient signed a written informed consent form before study enrollment. The trial is registered in ClinicalTrial.gov, identifier NCT04889586; date of registration: 17 May 2021. https://clinicaltrials.gov/ct2/results?term=NCT04889586 (accessed on 6 May 2021).

The inclusion criteria were: (1) diagnosis of first-ever cortical-subcortical unilateral, ischaemic, or hemorrhagic stroke, documented by computed tomography (CT) scan or structural magnetic resonance imaging (MRI); (2) older than 18 years. The exclusion criteria were: (1) presence of other neurological diseases as comorbidities; (2) not stabilized fractures; (3) severe apraxia, (4) severe neglect, (5) severe cognitive and communication impairment, and (6) untreated epilepsy, which were all assessed with the Oxford Cognitive Screen tool.

The following demographic characteristics were collected for each patient: age, sex, distance between stroke onset and clinical assessment (months), type of lesion (i.e., ischaemic, hemorrhagic), and hemisphere lesioned (i.e., right, left).

The study protocol was approved by the Ethics Committee for Clinical Experimentation (CESC) of Venice and San Camillo IRCCS hospital (Prot. No. “2016.29 MoRe” n. 836).

### 2.2. The Device

REMO^®^ is a wearable armband developed by Morecognition S.r.l. in collaboration with the Istituto Italiano di Tecnologia (Genoa, Italy) and the San Camillo IRCCS hospital (Venice, Italy). REMO^®^ is composed of 8 bipolar surface electrodes transmitting data to a host device (PC, tablet) via Bluetooth. REMO^®^ integrates an inertial measurement unit (IMU) enabled to standardize the armband position to unify the sEMG acquisition in all the subjects. The IMU is composed of an accelerometer, gyroscope, and a magnetometer. In this work, we did not analyze the kinematic movements: only data provided by the gyroscope were used to place the device on the patient’s forearm in the correct position.

REMO^®^ detected the total muscle activity (i.e., sEMG) of the forearm circumference with no need to detect the electromyography signal of a specific single muscle. In this way, the potential use of REMO^®^ device may reduce crosstalk and increase feasibility of the device in controlling the computer-interface for rehabilitation training in stroke survivors (Video S1: “REMO^®^ rehabilitation exercise”, Supplementary Materials). This strategy was inspired by prosthesis control development [37,38]. The detailed characteristics of the whole system were described in a previous work [35].

The participants wore REMO^®^ on the paretic forearm at a distance of 5 cm from the olecranon. To avoid the artefact noise to the sEMG signal caused by patient’s movements, the elbow was positioned on an arm support to prevent table hitting. The use of the arm support in the test setting did not interfere with the patients’ motor performance. Indeed, the arm support permitted the patients to move freely in all directions without any planar movements’ restriction.

**Figure 1:**
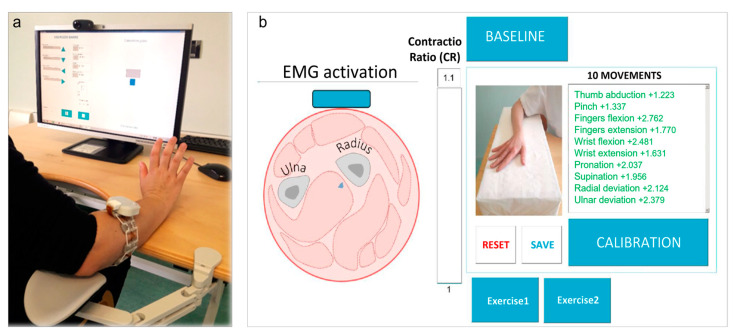
REMO^®^ Test Setting. (**a**) The patient wears REMO^®^ on the paretic forearm while seated at the height-adjustable ergonomic table. In this figure, the patient is performing an example of exercise for hand motor training. (**b**) Graphical user interface displaying real-time surface-electromyography (sEMG) amplitude on a radar graph with the list of movements to be tested and control buttons for sEMG-biofeedback training. The bar on the screen is the feedback provided to the patient referred to as the level of Contractio Ratio (CR).

Figure 1 shows the experimental setting for a representative subject: the computer-interface was developed to test REMO^®^ device as a controller of a rehabilitative virtual environment, based on surface-electromyography biofeedback training (Figure 1).

Thus, the tool included visual and auditive feedback that allowed the subjects to perform the tasks required. The visual feedback provided by the device is proportional to muscle activation: the stronger the muscle contraction, the larger the displacement of the pointer represented in the exercise. Moreover, the auditory feedback provided information about accomplishment of the task.

### 2.3. Test Methods

The study consisted of two assessment phases of participants: the clinical assessment of the upper limb motor function and the testing of REMO^®^ in performing 10 specific hand gestures.

Firstly, we clinically assessed the participant using standard clinical scales. Clinical assessment consisted of the following outcome measures: various sections of the Fugl-Meyer Assessment (FMA) scale (i.e., Upper Extremity, FMA-UE; wrist and hand sub-items, FMA-hand; sensation, FMA sensation; range of motion and pain, FMA pain/ROM), the Reaching Performance Scale (RPS), and the Box and Blocks Test (BBT). In addition, we used the Nine Hole Pegboard Test (NHPT) to assess hand dexterity of the stroke-affected side. We considered the Modified Ashworth Scale (MAS) to assess the level of spasticity of 5 muscles in the affected limb: Pectoralis Major (PecMaj), Biceps Brachii (BicBra), Flexor Carpi muscles (FlexCarp), Flexor Digitorum Profundus (FlexDigProf), and Flexor Digitorum Superficialis (FlexDigSup). Finally, we used the Functional Independence Measure (FIM) to assess independence in activities of daily living.

**Figure 2:**
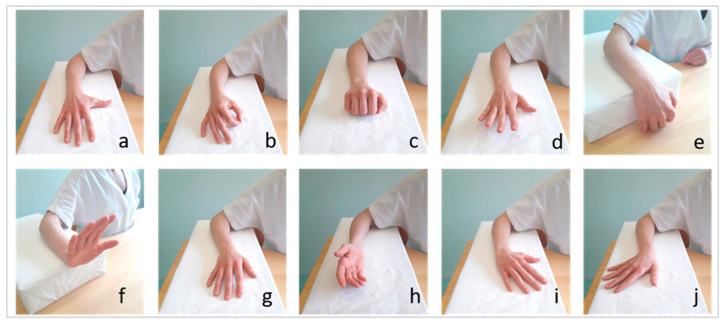
The ten movements tested by REMO^®^ In The Experimental Setting. Thumb abduction (**a**), pinch (**b**), fingers flexion (**c**), fingers extension (**d**), wrist flexion (**e**), wrist extension (**f**), pronation (**g**), supination (**h**), radial deviation (**i**), and ulnar wrist deviation (**j**).

Then, to test the use of REMO^®^ in performing 10 specific hand gestures, we asked the participants to perform 10 specific hand gestures with their hemiparetic hand. Hence, we recorded the muscle pattern associated with each of the following 10 hand gestures: thumb abduction, pinch, finger flexion, finger extension, wrist flexion, wrist extension, forearm pronation, forearm supination, radial wrist deviation, and ulnar wrist deviation (Figure 2).

The test with REMO^®^ consisted of two sub-steps: first, the subject was asked to leave the hemiparetic hand relaxed on the table for three seconds, so that REMO^®^ could record the baseline activity at rest of the forearm muscles. Then, the subject performed the maximum voluntary contraction (MVC) of the 10 gestures one by one and each pattern of muscle activation was recorded by REMO^®^ (i.e., sEMG) for three seconds. We required the gestures in the same sequence for each participant, as we showed in Figure 2. The subject had three possibilities to perform each movement. Between the performance of two different gestures, we asked the patient to relax the hand for one minute.

The test of REMO^®^ defined the number of gestures that the patient was able to perform to control the PC interface. Thus, we imposed that a subject was considered able to control a gesture if the ratio between the sEMG detected during the gesture MVC was higher than 10% of sEMG detected while resting at baseline. We define the threshold of control ability of a gesture as the Contraction Ratio (CR). Moreover, the PC interface provided positive feedback to the patient when he overcame the CR, represented by a fully colored bar (Figure 1). The threshold level was imposed to 10% based on our latest work, in which we defined the level of muscle activity needed to control a robotic device with sEMG wearable control modality [39].

### 2.4. Statistical Analysis

Data were collected at the Laboratory of Rehabilitation Technologies of the San Camillo IRCCS hospital (Venice, Italy) and analyzed using Rcmdr software [40]. Descriptive statistics of the sample were reported as mean and standard deviation (SD).

To determine the sample size, we considered recent evidence on the possibility of detecting muscle activity at the forearm, meant as voluntary control of sEMG amplitude since the first week after stroke, also in those patients not expressing any active voluntary movement [29]. Moreover, for these impaired patients, more than 90% of contractions involved at least one muscle. Thus, in our study, we expected that at least 90% of screened patients would be able to express valid sEMG signals to control the device, or else making the device able to recognize several patterns of sEMG activation. Therefore, considering the expected frequency, an alpha error of 0.05 and a statistical power of 90%, we calculated that a sample size of at least 97.4 subjects guaranteed the expected statistical power.

To predict the ability of using REMO^®^ in three different conditions of ability (i.e., 10, 5 or 0 hand movements controlled), we hypothesized a priori three different conditions: (i) no. movement controlled = 0, (ii) no. movements controlled = 5, (iii) no. movements controlled = 10. Furthermore, we explored the responses in the population by cluster analysis to validate our sample stratification based on a-priori numbers of gestures controlled. We adopted K-means as supervised clustering analysis and the number of a-priori clusters was set equal to 2. K-means is based on an algorithm approach for clustering analysis, allowing the partition of data collected into k cluster by identifying the centroid vector of the nearest means for the cluster [41,42]. K-means cluster analysis was conducted using Matlab version 2018a (The MathWorks, Natick, MA, USA) with the following parameters: the number of a-priori k clusters was set equal to 2, squared Euclidean distance was used to minimize within-cluster distance, and 100 maximum number of iterations. The algorithm returned an index that corresponded with the cluster that the observation was assigned.

Therefore, for each strata of ability to control REMO^®^, we estimated logistic multivariate regression models (i.e., GLM0, GLM5, GLM10, GLMk) to predict patients’ demo-graphic and clinical features, using a dependent variable as the test condition (i.e., number of movements performed with CR higher than 10%) and independent variables as the demographic characteristics (i.e., age, sex), disease conditions (i.e., months from lesion, type of lesion, side affected, presence of aphasia or apraxia), and outcome measures of the patients. In addition, the goodness of fit of each regression model was evaluated using the following indices [43]: the McFadden index of explained variance (pseudo-R^2) [44] and the Scaled Brier Score (sBS), which is a measure of overall accuracy and calculates the mean prediction error [45].

For each regression model, we evaluated the accuracy of the obtained cut-off by (i) area under the curve (AUC); (ii) calculation of sensitivity (se) and specificity (sp); (iii) confounding matrix or misclassification table; and (iv) overall classification accuracy (acc). The cut-off value was considered optimal if the indices met the following criteria: (i) AUC values > 0.70; (ii) best possible balance between sensitivity and specificity; (iii) low misclassification error rate; and (iv) highest possible accuracy index (acc).

Thus, the Receiver Operating Characteristic (RoC) curve was used to establish the clinical outcomes’ cut-off (k) able to predict the ability of controlling the device. Odds Ratio and their 95% confidence interval (CI) were computed. Finally, polyserial correlation (r) between the significant patient characteristics (demographic and clinical) and the numbers of movements executed by the patients were calculated. Statistical significance level was set at *p* < 0.05.

## 3. Results

Overall, 117 patients were enrolled in the study. All subjects completed both the clinical and the instrumental assessment tests, and no adverse events were reported. All patients declared that the device was easy to wear and that the computer-interface was user-friendly. The demographics and clinical characteristics of the sample are reported in Table 1.

After the test, 76 subjects (65%) were able to control all the movements, 22 subjects (19%) were able to control a portion of movements, and 19 subjects (16%) were able to control no movement. By exploring clinical features of patients allocated in the three groups, we observed differences in the FMA-hand average scores between the three groups of patients controlling all the gestures (14.29 ± 8.06 points, 9 to 1 movement (3.45 ± 6.92 points) and those unable to control any movement (0.05 ± 0.23 points).

**Figure 3:**
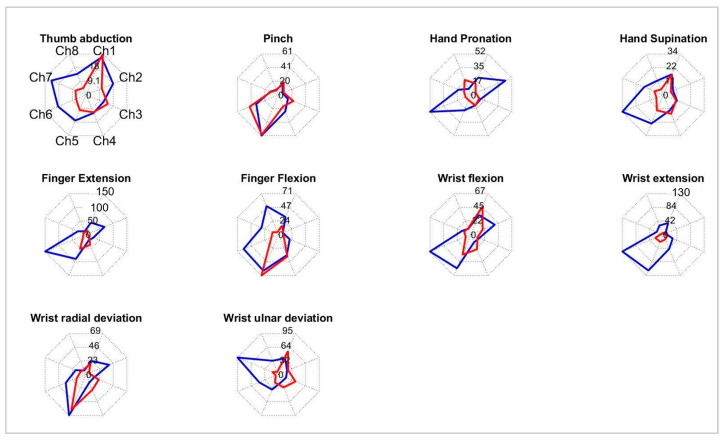
Example of radar graph representation of ten movements’ muscle activations of two patients (Blue and Red line). Each graph is scaled according to the maximum channel output of the two patients.

Moreover, in the subgroup of patients able to partly control REMO^®^ (*n* = 22), the frequencies of patients able to perform the gestures were the following: finger extension and finger flexion were performed by 16 subjects (72.7%), wrist flexion and radial deviation by 15 subjects (69.2%), wrist extension and ulnar deviation by 12 subjects (54.5%), pronation by 11 subjects (50%), supination by 10 subjects (45.5%), pinch by 9 subjects (40.9%), and thumb abduction by 7 subjects (31.8%). Moreover, we observed differences in the muscle activation of ten movements. Figure 3 shows an example of sEMG representation of two patients’ movements’ performances (Figure 3).

To define which variables were associated with the three different conditions of control, three multivariate logistic regression models were built. The results showed that only the clinical features of the patients influenced the ability to execute the instrumental test with the REMO^®^ device. Indeed, no demographic characteristics and no disease conditions influenced the results of the regression models.

**Figure 4:**
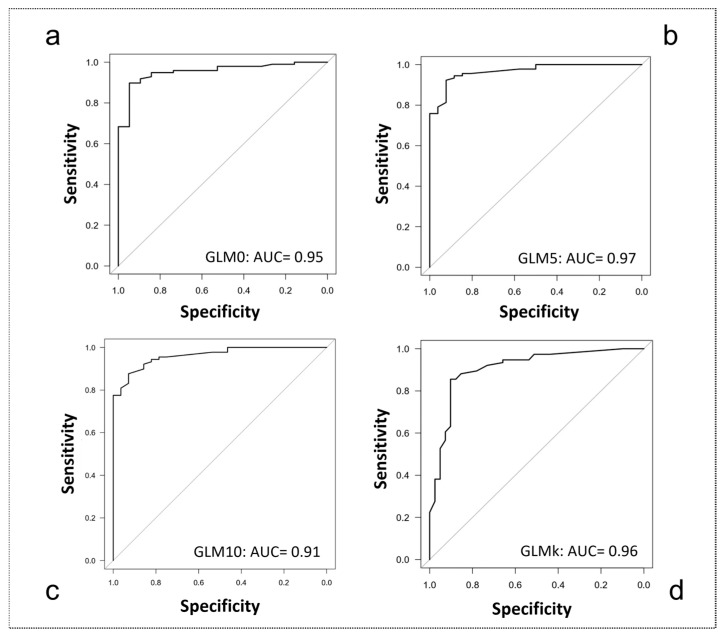
Area under the Curve (AUC) graphs for classification accuracy. (**a**) 0 movements controlled (GLM0); (**b**) up to 5 movements controlled (GLM5); (**c**) up to 10 movements controlled (GLM10); (**d**) k-means classification (GLMk).

The ability to execute at least one movement (GLM0) was predicted by the following outcome measures, with an accuracy of 95%: FMA-UE (coefficient: 0.42; Z = 3.22; *p* = 0.001), FMA pain/ROM (coefficient: 0.20; Z = 2.48; *p* = 0.013). The ability to execute at least five movements (GLM5) was predicted by the following outcome measures, with an accuracy of 97%: FMA-UE (coefficient: 0.50; Z = 3.28; *p* = 0.001), MAS of Flexor Carpi muscles (coefficient: −0.79; Z = −2.20; *p* = 0.028). Finally, the ability to execute all ten movements (GLM10) was predicted by the following outcome measures, with an accuracy of 91%: FMA-UE (coefficient: 1.10; Z = 5.28; *p* = 0.000). The AUC curves of the logistic regression models are displayed in Figure 4.

**Figure 5:**
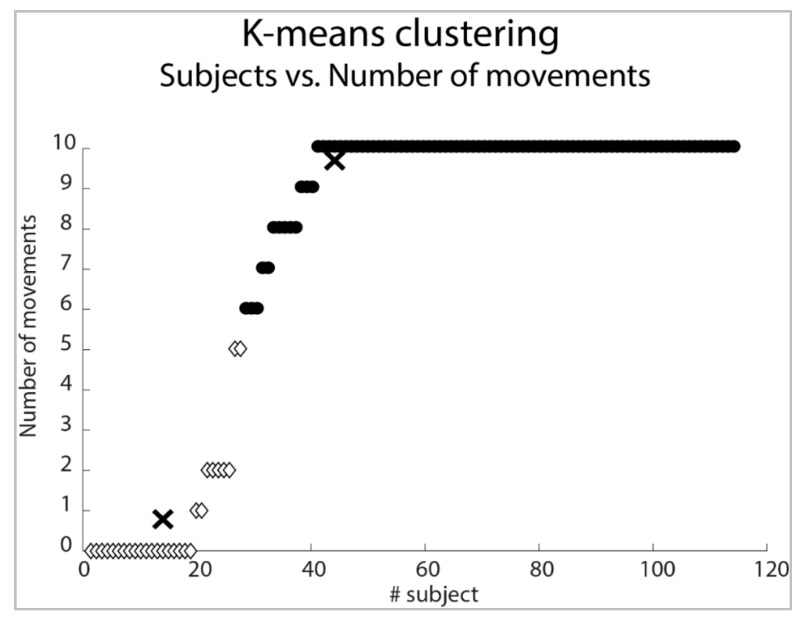
Subjects classification (K-Means) according to the number of movements tested. In the picture, X-signs correspond to k centroid of each cluster. Two clusters are shown with white diamonds and black filled points.

The K-means cluster analysis distinguished two groups: a first group including patients able to control up to five movements (k centroid = 0.78) and a second group including patients able to control more than six movements (k centroid = 9.60) (Figure 5).

According to this separation, we implemented a fourth regression model (GLMk) to detect the outcome measure able to predict the ability to control more than six movements: FMA-UE (coefficient: 0.42; Z = 3.20; *p* = 0.001) and MAS at the level of Flexor Carpi muscles (FlexCarp coefficient: −0.67; Z = −2.03; *p* = 0.043).

Accuracy of the GLMk model was 96%. Finally, Odds Ratio and cut-offs (k) of the clinical outcome measures retrieved for each logistic regression model were calculated.

In Table 2, we reported the characteristics of all GLM models and relative Odds Ratio values.

Finally, polyserial correlation between the FMA-UE points and the number of movements executed by the patients for all three models was r = 0.80 (*p* < 0.01).

## 4. Discussion

In our study, we aimed to define clinical features in order to use a sEMG wearable to control a computer-interface developed for hand rehabilitation of stroke survivors. In the test, the patients were asked to execute ten different hand movements wearing the device REMO^®^. We defined a priori three different conditions of control (i.e., ten, five, zero movements) to classify the patients based on the number of hand movements performed with REMO^®^ and to investigate the clinical features of each strata of patients.

A first result was that most of the tested patients (65%) were able to control all ten of the requested movements. Furthermore, an adjunctive portion of patients (19%) was able to control part of the movements (i.e., from one to nine), demonstrating device flexibility in gesture recognition, which makes REMO^®^ adaptable to many different functional conditions in stroke survivors. In subjects able to control the device, gross motor movements of the hand (i.e., fingers flexion and extension) were the easiest to perform, whereas thumb abduction was the most difficult task to be recognized. Our data confirmed literature evidence of classification of hand gestures by sEMG pattern recognition, demonstrating that wrist and fingers’ flexion/extension are classified more accurately than finger singularization and functional grasps [46,47]. Furthermore, difficulty in classifying thumb abduction in our sample confirmed findings from Carpinella et al. reporting impairment of inter-digit coordination during thumb motion after stroke [48]. In our previous work, we tested with REMO^®^ the same hand gestures required in this paper but coupled in five functional couples. The results showed that the most impaired movements for the patients were thumb abduction and pinch, in which they needed more time to perform the muscle activation with the hemiparetic hand [36]. Moreover, the results suggested that the training with REMO^®^ induced an improvement of motor performance. Indeed, the patients’ muscle activations were more precise and fast in all movements required, which may be correlated with an improvement of motor control [36].

In the clinical features investigation of the tested patients, results indicated that to refer a patient to treatment with REMO^®^, the upper limb sensory-motor functions needed to be assessed are: motricity, spasticity at the Flexor Carpi muscles, and pain and joints ROM. None of the demographic conditions influenced the ability to control the device. Thus, any differences about age, sex, type of lesion, side of lesion, the presence of apraxia or aphasia, and time from lesion conditioned the use of REMO^®^. Therefore, only the clinical features indicated the level of ability to control the device, thus the possibility to be referred to a specific technological-based rehabilitation treatment for hand motor recovery (i.e., sEMG-biofeedback and virtual reality training).

The first main result is that patients with a severe impairment of the upper limb (FMA-UE < 10/66 points) cannot control the armband, and thus should not be referred to treatment with such a device. Conversely, to control at least one movement or being able to meaningfully interact with an external (artificial) environment, a minimum of ten points at the FMA-UE with negligible pain and joints restriction at the upper limb (FMA-UE pain/ROM ≥ 43/48) should be registered. Our results reflected the difficulty of muscle activation in patients with a higher level of upper limb motor impairment. Papazian et al. affirmed that in severely impaired patients, there were upper limb muscle contractions without visible movements, but they reflected random and uncontrolled muscle activity [29]. However, sEMG-driven interventions should be feasible to this type of patient when considering the limited rehabilitation options available for people with severe upper limb impairments [29]. These data suggested that more investigation of sEMG-driven intervention of patients with severe motor impairment is needed.

The empirical cut-off distinguishing the ability to control a small (i.e., <5 gestures) or a large (i.e., >5 gestures) number of movements was independently confirmed by cluster analysis that set the computational threshold at six, with the same set and cut-off of predictive outcome measures. Thus, to control up to six gestures, patients need some residual motor function (FMA-UE ≥ 18/66 points), together with the absence of spasticity at the flexor carpi muscles (Flex Carp < 0/4 points). These results confirm the findings from Meerker et al. who reported that hypertonia affects the ability to control external devices by sEMG. In fact, subjects expressing hypertonia at the flexor muscles of the wrist may have difficulty in relaxing the hand after voluntary flexion to recruit the extensor muscles of the wrist [34]. Finally, full, independent, and autonomous control of the armband (i.e., 10 gestures) is possible for patients with severe-mild impairment of the upper limb (FMA-UE ≥ 18/66 points).

In summary, the administration of some parts of the Fugl–Meyer Assessment (i.e., FMA-UE, sensation, and pain/ROM section) and the Modified Ashworth Scale at wrist flexor muscles was sufficient to accurately predict the number of gestures a patient was able to intentionally control at the level of sEMG signals. These findings are consistent with previous studies from Van Kordelaar et al. who claimed the clinical relevance of the Fugl-Meyer Assessment scale because it reflects the patient’s overall ability to involve the upper limb in rehabilitative training [49].

From the physical therapist perspective, the possibility to tailor the training (i.e., type or number of tasks) to the clinical features of patients is in line with recent recommendations [50]. Furthermore, this possibility is an added value provided by innovative technology able to detect voluntary behaviors (i.e., automatic movement detection) [28]. Therefore, similar approaches have been employed for prosthetic control, where customizable calibration allows for personalization and expansion of the device control according to the patient’s needs and capabilities [51].

Finally, usability and customization of REMO^®^ are fundamental advantages for future applications. The advantage of the wearable device reduced the problem of crosstalk, artefacts, and noise from the electrical current supply [52]. These characteristics of REMO^®^ allow for use in a neurorehabilitation setting, such as home-based rehabilitation or task-specific training with real-time sEMG biofeedback. Moreover, the device provided monitoring of muscle activation patterns, allowing for the monitoring of motor recovery of stroke survivors during the whole recovery process.

Our study is affected by several limitations. A current limit is the lack of a validated model for movements classification using REMO^®^. In fact, in this work, we did not investigate the pattern recognition of each gesture to classify the patients tested. However, our data confirm that there is a strong positive correlation (r = 0.80, *p* < 0.01) between preservation of upper limb motor function and the number of movements performed by patients using the device. Moreover, future investigations will be performed to classify stroke patients based on the normal muscle activation model (i.e., healthy subjects) of hand gestures.

Another current limit is to determine the REMO^®^ capability of classifying a defined number of hand movements. This limitation is due to the technical design of the armband. In fact, it is acknowledged that the number of Degree of Freedom (DoF) of a device represents a physical limit for its active control [53,54]. In this regard, in our experimental test, patients were asked to perform ten different hand gestures, despite REMO^®^ being composed of eight electrodes, which represents the maximum number of elements that the device is made of [55,56]. In healthy subjects, the accuracy of pattern recognition from sEMG decreases with the augment of the number of gestures to be recognized [57,58]. In stroke patients, the accuracy of pattern recognition from sEMG decreases even within the repetition of the same gestures, due to phenomena such as fatigue or weakness [59]. Thus, REMO^®^ solution, based on multi-channel sEMG signals, may represent a powerful option to obtain good performance of movement classification, also in pathological conditions.

## 5. Conclusions

In this study, we stratified a sample of stroke patients according to their sensory-motor profiles to predict their abilities to control a computer-interface using a sEMG wearable armband. In this way, we retrieved information for customization of innovative sEMG-based training provided by REMO^®^. Indeed, most stroke survivors may use REMO^®^ at its maximum possibilities to train their hand motor function in order to enhance motor recovery after stroke. To test the efficacy of using REMO^®^ as biofeedback during functional training, a pilot clinical trial has been designed.

## Figures and Tables

**Table 1:** Demographic Characteristics of Patients.

Age	
Mean Years (SD)	65.09 (12.14)
Sex	
Female/Male, *n* (%)	44/73 (38/62%)
Aphasia	
Yes/No, *n* (%)	37/80 (32/68%)
Apraxia	
Yes/No, *n* (%)	7/110 (6/94%)
Lesion Type	
Ischemic Stroke, *n* (%)	77 (66%)
Hemorrhagic Stroke, *n* (%)	40 (34%)
Affected arm	
Right/Left, *n* (%)	56/61 (48/52%)
Time from stroke	
Mean months (SD)	15.98 (35)
FMA, Mean (SD)	
FMA-UE	30.04 (22.66)
FMA-hand	9.94 (9.33)
FMA sensation	16.44 (7.99)
FMA Pain/ROM	42.40 (5.28)
RPS	
Mean (SD)	16.62 (14.91)
NHPT	
Mean (SD)	0.13 (0.20)
BBT	
Mean (SD)	14.82 (18.97)
FIM	
Mean (SD)	85.95 (24.37)
MAS, Mean (SD)	
Total score	1.92 (2.83)
PecMaj	0.29 (0.68)
BicBra	0.49 (0.75)
FlexCarp	0.56 (0.93)
FlexDigProf	0.24 (0.65)
FlexDigSup	0.35 (0.69)

Data are reported as Mean, Standard deviation (SD), and percentage. FMA-UE: Fugl–Meyer Upper Extremity; FMA-hand: wrist and hand motor sections; FMA sensation: Fugl–Meyer sensation; FMA pain/ROM: Fugl–Meyer pain and joint range of motion; RPS: Reaching Performance Scale; NHPT: Nine Hole Pegboard Test; BBT: Box and Blocks Test; FIM: Functional Independence Measure; MAS: Modified Ashworth Scale total score; PecMaj: Pectoralis Major; BicBra: Biceps Brachii; FlexCarp: Flexor Carpi; FlexDigProf: Flexor Digitorum Profundus; FlexDigSup: Flexor Digitorum Superficialis.

**Table 2:** Classification of Subjects According to Clinical Outcome Measures.

Model	N Movements	Variables	Odds Ratio (CI 95%)	K Value/Total	Se/Sp
GLM0	N > 0	FMA-UE	1.53 (1.18–1.98)	≥10/66	0.95/0.82
		FMA-UE pain/ROM	1.22 (1.04–1.43)	≥43/48	0.79/0.65
GLM5	N ≥ 5	FMA-UE	1.65 (1.22–2.22)	≥18/66	1/0.76
		FlexCarp	0.45 (0.26–0.92)	<0/4	1/0
GLMk	N ≥ 6	FMA-UE	1.52 (1.18–1.97)	≥18/66	1/0.76
		FlexCarp	0.51 (0.26–0.98)	<0/4	1/0
GLM10	N = 10	FMA-UE	1.11 (1.07–1.15)	≥18/66	0.90/0.86

Significant outcome measures predicting ability to control REMO^®^ for each of the device control conditions. CI: 95% confidence interval; k value/total: the cut-off score over the total score of the outcome measure; Se/Sp: sensibility/specificity.

## Data Availability

The datasets used and analyzed during the current study are available from the corresponding author on reasonable request.

## Supplementary Materials

The following supporting information can be downloaded at: https://www.mdpi.com/article/10.3390/ijerph20065082/s1, Video S1: REMO^®^ rehabilitation exercise.

## Abbreviations

TBR	Technology-Based Rehabilitation
sEMG	Surface Electromyography
CT	Computed Tomography
MRI	Magnetic Resonance Imaging
IMU	Inertial Measurement Unit
CR	Contraction Ratio
MVC	Maximum Voluntary Contraction
FMA	Fugl-Meyer Assessment scale
FMA-UE	Fugl-Meyer Assessment Upper Extremity
FMA-hand	Fugl-Meyer Assessment Upper Extremity, wrist and hand sub-items
FMA sensation	Fugl-Meyer Assessment, sensation section
FMA pain/ROM	Fugl-Meyer Assessment, range of motion and pain section
RPS	Reaching Performance Scale
BBT	Box and Blocks Test
NHPT	Nine Hole Pegboard Test
MAS	Modified Ashworth Scale
PecMaj	Pectoralis Major
BicBra	Biceps Brachii
FlexCarp	Flexor Carpi muscles
FlexDigProf	Flexor Digitorum Profundus
FlexDigSup	Flexor Digitorum Superficialis
FIM	Functional Independence Measure
AUC	Area Under the Curve
RoC	Receiver Operating Characteristic
CI	confidence interval
GLM	logistic multivariable regression model
GLM0	logistic multivariable regression model, 0 movement
GLM5	logistic multivariable regression model, 5 movements
GLM10	logistic multivariable regression model, 10 movements
GLMk	logistic multivariable regression model, k-means output number
DoF	Degree of Freedom
